# Filial piety and parental responsibility: an interpretive phenomenological study of family caregiving for a person with mental illness among Korean immigrants

**DOI:** 10.1186/1472-6955-11-28

**Published:** 2012-12-20

**Authors:** Mijung Park

**Affiliations:** 1 Department of Health and Community Systems, University of Pittsburgh School of Nursing, 3500 Victoria Street, 421 Victoria Building, Pittsburgh, PA 15261, USA

**Keywords:** Family caregiving, Immigrant family, Korean Americans, Confucianism

## Abstract

**Background:**

Despite the strong influence of culture on family involvement in disease management, few studies have examined how immigrant families care for persons with mental illness. The purpose of this study was to examine how immigrant families organize their world to care for a mentally ill person in the United States. The current analysis focused on how Confucian notions of filial piety and parental obligation shape caregiving in Korean immigrant families.

**Methods:**

Participants in this interpretive phenomenological study were comprised of six Korean immigrant women caring for a family member with mental illness. Participants provided narratives that illustrate challenges and opportunities in caring for their mentally ill family member.

**Results:**

Three family caregiving patterns were discerned. Insulating from the outside world describes a family’s effort to accept a member's illness and to manage it within the family. Prioritizing education over well-being concerns parental commitment to the Confucian priority of educating one’s children. Reciprocating the sacrifice describes how a family adapts and enacts filial piety.

**Conclusion:**

The findings of this study warrant further study to examine the influence of Confucianism among Korean American families. The three patterns of caregiving are strongly aligned with Confucian notion of family and family engagement. These patterns may help health providers to anticipate the needs of and provide individualized, culturally appropriate mental health care for patients with mental illness and their families of Korean origin.

## Introduction

As the majority of developed countries become more racially and ethnically diverse, the capacity of health care systems to respond to patients’ varying perspectives, values, and needs regarding health and illness becomes increasingly important. Nurses working with multicultural patients have long grappled with the notion of ‘culture’ and its implications for clinical nursing practice. The culture often defines what constitutes a mental illness and what may be done about it. Culture also shapes the manner and degree of family involvement in treatment of mental illness. Some cultural groups may expect the patient’s family to be included in decision-making regarding treatment [1,2]; others may consider such decisions typically up to the patient. Nurses should be able to navigate these complex expectations and social mores related to health behavior when caring for culturally diverse patients. Unfortunately, few studies exist to guide nurses when they encounter Korean immigrants with mental illness and their families. The purpose of this paper is to examine whether Confucian notions of filial piety and parental obligation are still present in modern Korean immigrant families and how these notions shape family management of mental illness. Confucianism was chosen as the theoretical foreground of this study because it has been the most influential force in shaping Korean family life [3].

## Background

### Disparities in mental health among Korean Americans is a serious problem

Korean Americans are one of the fastest growing ethnic minorities in the United States. The Census 2010 shows that Korean American population has grown from 354,529 in 1980 to 1.42 million in 2010 [4], comprising approximately 11% of the Asian American population. Compared with other ethnic groups, they are newer immigrants; about 80% of Korean Americans are foreign-born [5]. Despite this rapid population increase, there is a significant paucity of research in this population [6]. The model minority stereotype that characterizes Asian Americans as relatively free from stressors or problems has been blamed for this void of study [7,8]. Contrary to this stereotype, the evidence of disparities in mental health among Korean Americans is clear and convincing. Korean Americans have (a) poorer mental health compared with Americans of Europeans or of other Asian descents, (b) fewer treatment options due to the lack of culturally and linguistically appropriate mental health care services, (c) the least insurance coverage and mental health service utilization, and (d) consistently report the greatest dissatisfaction with health care services of all racial and ethnic groups [9-14]. There is an urgent need to eliminate the health disparities in this vulnerable population.

### Family caregiving is culturally grounded and culturally prescribed

The family context has powerful effects on management of and service utilization for mental illness [15,16]. The evidence is compelling that family support is protective and beneficial to persons with a chronic illness [17-20]. Studies have consistently shown that individuals with strong family support are less likely to be placed in an institution and that the absence of family caregiving is a leading predictor of institutional placement [21].

Family support and caregiving is grounded in culture and varies among cultural groups. However, there is limited research on how culturally unique family caregiving affects individual health outcome in minorities such as Korean Americans [22]. Understanding culturally unique family caregiving in Korean Americans may be particularly important because of their collectivistic social orientation and emphasis on hands-on caregiving [23]. This lack of knowledge hinders culturally appropriate mental health treatment and contributes to health disparities in this population.

Strong evidence suggest that Asian Americans are often intensely engaged in the illness management of a family member; therefore understanding their unique family approach to mental illness is particularly important [24]. Compared to their White counterparts, Asian American families provide more hours of care with fewer resources but with more informal support [25-27]. This strong family care is consistent among Korean Americans. Disproportionately high rates of Korean Americans with mental illness live with, or receive regular care from their family. In one study, 95% of Korean Americans with schizophrenia live with their family compared with only 9.5% of their White counterparts [28].

Three reasons may explain why hands-on family caregiving is so pervasive among Korean Americans. First, a family may feel cultural pressure to provide hands-on caregiving for a family member. The strong emphasis on filial responsibility, family cohesion, and harmony over individual happiness still shapes family caregiving practices among modern Asian Americans [29]. Consequently, Korean Americans may be reluctant to place a chronically ill family member in a long-term health care institution, such as a nursing home, due to the negative connotations associated with such a decision [30]. Second, because of the stigma associated with mental illness, patients and families often delay treatment and suffer a poor prognosis [31-33]. Third, the lack of linguistically and culturally appropriate health care is largely responsible for delayed treatment and health care service disparities in this population. As a result, Korean Americans often assume 24-hour caregiving responsibility for a chronically ill family member [34,35], often eschew ambulatory health clinics for treatment [35,36] and use emergency departments [16,37].

### Confucianism provides a good conceptual framework to understand Korean American family caregiving

Confucianism has implicitly and explicitly governed various aspects of individual and family life, and plays a role in shaping the values and preferences related to treatment of mental illness in Korean society. Buddhism and Taoism have strongly influenced traditional Korean culture, and Christianity has become increasingly influential for modern Koreans and Korean Americans. However, Confucianism is particularly relevant to studying families of Asian origin, including Korean Americans [1,38], because of its pervasive and prolonged influence on Asian family life [1,38].

The core tenets of Confucianism emphasize the individual obligations towards family: being a loving parent, taking good care of offspring, being a dutiful child, and supporting siblings. These obligations are considered to be forms of self-realization. Educating a child is emphasized as a way of raising a good member of society and is regarded as the single most important parental obligation. Similarly, caring for one’s elderly parents is the primary moral mandate for all offspring [39].

Since the majority of Korean Americans are foreign-born and immigrated to the U.S. recently, studying their culture of origin will render meaningful understanding of this population. Furthermore, the culture of origin is a good starting point to better understand how immigrants negotiate the cultural differences between their culture of origin and that of the host society. However, the acculturative process of families may differ from that of individuals [40,41]. For example, third generation Japanese Americans retain some characteristics of the traditional Japanese family system [42]. This study finding suggests that, although significant acculturation may be occurring, the influence of cultural origin may extend well beyond the immigrant community.

## Methods

The aims of this interpretive phenomenological study were to examine whether Confucian notions of filial piety and parental obligation are still present in modern Korean immigrant families and to describe how these notions shape family management of mental illness as they acculturate to American society.

### Design

Interpretive phenomenology [43], which advocates the investigation of detailed personal narratives of actions and choices to distill habits and practices, guided the design and conduct of this study. Interpretive phenomenology is an ideal methodology to study family process because it acknowledges shared meaning among family members and takes familial commitments and values seriously [44].

### Sample

Purposeful sampling was used to recruit Korean Americans who have experienced caring for a mentally ill family member. Recruitment methods included advertisements; flyers posted in public places, such as clinics, markets, and churches, frequented by Korean Americans; and snowball techniques [45]. Inclusion criteria were (a) self-identified as a Korean American, (b) have a mentally ill family member who had been diagnosed at least one year before data collection, (c) aged 21 or older, and (d) resided with or had at least weekly contact with the mentally ill family member. Caretakers diagnosed with a mental illness were excluded from the study. None of the potential participants met this criterion.

A two-stage sampling strategy was used in this study. We first recruited, collected, and analyzed data with three participants to test recruitment strategies and analytical approaches. Three more participants were then recruited. Although the number of participants in the second stage was not pre-determined, after interviewing three participants it became clear that the Confucian notions of filial piety and parental obligation were present in all participants. At this point, we ended our recruitment.

The participants (*N* = 6) included four mothers, one daughter-in-law, and one sister. The average age of the participants was 52.8 years, ranging from 38 to 68 years. The average residence in the United States was 28 years, ranging from 4 to 40 years (see Table 1).

**Table 1:** Demographic Data of Participants (N = 6)

	**Caregiver**		**Patient**	
	Age*	**Relationship**	**Diagnosis**	**Age**
P1	Early 50s	Mother	Depression/ADHD	18
P2	Late 40s	Mother	Depression	16
P3	Late 50s	Daughter-in-law	Dementia	78
P4	Late 60s	Mother	Schizophrenia	18
P5	Late 50s	Mother	Schizophrenia	29
P6	Late 30s	Sister	Schizophrenia	18

Note: U.S. = United States; ADHD = Attention Deficit Hyperactivity Disorder Only caregivers participated in the study.

* The information about the age was provided in approximation to anonymise the information.

### Procedure

Data from this study consists of eight in-depth, face-to-face interviews with six participants between 2006 and 2007. Follow-up interviews with two participants were needed to confirm and clarify content of the first interviews. All interviews, including follow-up interviews, took no more than 90 minutes and were conducted at locations requested by each participant. Data was collected by a bilingual psychiatric nurse with 16 years of experience in Korea and in the U.S. Participants were asked to use either Korean or English as they wished in their interview.

The interviews were open-ended and minimally structured, focusing on the participants’ narratives of their habits and practices in caring for a mentally ill family member. Interviews started with a broad invitation, such as “Please tell me about your experience of caring for a family member.” Follow-up questions included narrative questions, such as “Can you tell me about specific incidents or a time that can illustrate what you just said?” and reflective questions, such as “Why do you think your approach worked?” Minimal prompts were used during the interviews to allow each person’s story to unfold [46].

Interviews were digitally recorded with the participants’ informed consent. Each interview was transcribed verbatim in the language, English or Korean, which was used to conduct the interview. During the transcription processes, unique identifiers were assigned to the participants to ensure their anonymity. In addition to face-to-face interviews, interpretive memos and field notes, written during and after the interviews, were included in the analysis. These field notes provided details about the context and flow of the interviews, the interviewer’s initial interpretations of what was said, and her reflections on each interview.

The research study was approved by the University of California San Francisco Committee on Human Research. Written informed consent for participation in the study was obtained from participants prior to starting each interview.

### Data analysis

Data analysis began after the first interview was conducted and extended through the final interpretation and articulation of the research findings. Analysis was guided by the study’s research aims and openness to learning about new and important concerns from the participants. We began the analysis of each case by reading the transcription of an interview several times. This was followed by a detailed page-by-page and narrative-by-narrative interpretation of the case. Additionally, each interview was constantly compared and contrasted with other interviews to deepen our understanding of the participants’ experiences.

The unit of analysis of this study was the narrative, a story organized around concrete and consequential events [47]. We focused on the context and background of an event, the action as it unfolded, and the emotional tone of the narrative. Thematic analysis was used to articulate the broader understanding that arises from constant comparisons of cases and narratives [46]. Once a theme was discerned, the research team examined how participants’ choices and actions varied by circumstances and contexts.

The interpretive team consisted of four members: this author and three doctorally prepared colleagues: a bilingual, Korean American nurse researcher and two American nurse researchers of European origin. Data were analyzed in the same language as the interviews were conducted and transcribed to retain the subtle nuances that might be lost by translating verbatim transcriptions into English. Parts of interviews were translated into English, to facilitate the interpretive process and to present study findings. ATLAS-ti, a computer software program, was used to organize and manage the data and analyses.

## Results

Three family caregiving patterns were discerned: *Insulating from the outside world, prioritizing education over well-being, and reciprocating the sacrifice.* Insulating from the outside world and prioritizing education over well-being describe how parents organized their world to cope with their child’s mental illness; reciprocating the sacrifice describes how adult children adapted the Confucian-inspired notion of filial piety. *Insulating from the outside world* describes the family’s approach of accepting a member’s illness and managing it within the family, whereas prioritizing education over well-being refers to families who embrace the Confucian notion of parental obligations and make the education of the child their primary goal.

### Insulating from the outside world

Insulating from the outside world describes the effort of a family to accept a member’s illness and to manage it within the family. This effort does not seem to be an aversion to stigma or shame. Rather, a family feels deep sorrow about a member’s illness, accepts the chronic nature of the illness, worries about the stigma and hardship the member may experience in the outside world, and tries to provide him or her with safe sanctuary. They thus manage the family member’s illness by insulating him or her from the stressors of the outside world. Among the six participants, three demonstrated this management style: a mother, a sister, and a single mother.

How the Q family cared for their son illustrates the notion of insulating from the outside world. The Qs decided to emigrate to the United States when their son (BQ) experienced difficulties in school and was subsequently diagnosed with schizophrenia. They hoped to find a more accepting environment for his problems in the U.S. When he began having problems in school again in the U.S., the parents decided to home-school him to prevent his being labeled a “failure”.

Mrs. Q: My husband was not enthusiastic when I started talking about homeschooling B (her son). My husband would tell me to let him do whatever he wished and to stop focusing on education. But, he agreed to stop sending B to school before he was kicked out again. Multiple failures in school are not a great record to have.

Mr. Q had resigned from his managerial job in Korea, took a blue-collar job in the U.S. to support his family, and later opened a small gift shop. Although it has been 4 years since the family moved to America, Mrs. Q still “felt that somehow unsettled in the U.S”. Even though B was 16 years old, Mrs. Q described him as if he were a little boy. In the small world that they created in the gift shop, their son was safe and cozy.

Mrs. Q: He seems O.K. in the shop. It is quiet and not many people bother him. He reads books, plays game station, and helps us cleaning. He’s very good at it. He does the cashier sometimes. He gets anxious and start making noises when he is around with lots of people, so we rarely go to public places anymore. We go to church and he knows everyone, so he does not get too anxious there.

A similar approach was discerned from the story of Mrs. K, a single mother caring for a daughter diagnosed with schizophrenia. Mrs. K owned two businesses: a newspaper stand and a clothing alteration shop. She took her daughter, who was in her mid-30s at the time of the study, to work each day. Mrs. K described her daughter’s activity as follows:

Mrs. K: When she (her daughter) was not in a “good mood”, she would lay down on the newsstand floor, withdrawn all day. Otherwise, she would help me selling newspaper.

By framing her daughter’s activity as “helping”, she conveyed that her daughter was a contributing member of society. The stigma of mental illness did not appear to concern Mrs. K because she openly discussed her daughter’s illness with her friends and church fellows.

### Prioritizing education over well-being

Prioritizing education over well-being describes how parents embraced the Confucian priority of parental obligations: the education of one’s child. The parents viewed education as the only means to ensure their child’s self-sufficiency in the future. They seemed unable to modify their expectations for their child, despite his or her mental illness, and insisted that he or she earn a diploma or an academic degree. New care needs seemed to add to their already heavy burden. They frequently experienced disappointment and frustration in the process. They also reported having constant conflict with their child. To ensure their child’s academic success, parents aggressively sought support from their church, school, and local social service agency. Two single mothers showed this caregiving pattern.

Ms. S was a single mother caring for an 18-year-old son diagnosed with schizophrenia. When her divorce was finalized, she took custody of her daughter, and her ex-husband took the son. But when the ex-husband reported that the son had been skipping school, she decided to intervene and eventually took full custody of both children. She worked at night in a post office. With many responsibilities and little support, her life was always hectic and tiring. Making sure that her son went to school taxed her time and energy significantly.

Ms. S: After working night shift, I had to pick him up from home, dropped him off at the school, and pick him up after school. I slept in between, and had to catch up with all the house chores.

Despite Ms. S’s dedication, the relationship between her and her son was rocky. Their constant arguing about school attendance and school assignments led to disappointment and frustration on both sides. Because her son’s graduation was *the* paramount goal, Ms. S channeled her limited energy to its achievement while overlooking other areas such as how her son felt.

Ms. S: When I came home from work in the morning after the 8-hour night shift, the house was a mess with screws and electric wires on the floor. He could unscrew things but unable to reassemble them. So we had to buy lot of things again and again. I was angry. I was very tired but have to clean this whole mess. And the things that you need were destroyed and you have to buy them again. I was just angry. I yelled at him. But he had to go to school in the morning. He had a hard time to wake up because he hadn’t sleep at night. So I had to wake him up. I did not have a good chance to talk with him.

Although Ms. S was upset that her son constantly made a mess of the house, she did not confront him about his behavior. Instead of doing so, Ms. S dropped him off at school without comment. Yet, the bitter feeling of anger and disappointment lingered.

Ms. S reported that she visited his school often and demanded more assistance for her son. She constantly juggled new tasks with the old. Her approach toward her son’s care was reactive rather than carefully planned. For example, when the school psychologist recommended a specialist to assess her son’s attention deficit hyperactivity disorder, she assumed another task.

Ms. S: We had to go to the specialist once a week. Once we arrived at the office, I could not drop him off like in school. I had to wait until his session was over. It was so tiring. But I had to do it. He has to graduate at least high school.

She enlisted the help of church members to tutor and mentor her son. In fact, youth group members visited her son daily and tutored him throughout high school, which enabled the son to graduate. Ms. S shared her hope that her son would enlist in the military, which might provide more structure in his life.

Unlike Ms. S’s struggle to keep her mentally ill son in the community, Ms. L accepted institutionalization and social services to provide her son with an educational opportunity. After the son’s many hospital admissions, a psychiatrist recommended a residential program for adolescents that offered schooling.

Ms. L: I didn’t know anything about mental illness. So, when his (the son’s) doctor recommended a residential program and told me that he could complete high school there, I though it was a very good opportunity for my son. At least he could complete and earn his diploma there. He is a man. He needs to have at least a high school diploma. Or else, how can he find a job? How can he survive in this harsh world?

During the son’s treatment, Ms. L reported feeling ambivalent about having her son at home on weekends because she could not help him with his homework. She reported that they argued about homework “all the time, because he watched television too much”. Ms. L wished that her son could enter a residential program for adults after high school graduation so that he could attend community college.

Ms. L: I want him to go to community college. It’s better than staying home and doing nothing with a high school diploma. But, my son’s case manager told me that college might be too much for him. She said that he does not have enough skills to handle the stress. I think if he could go to another residential program, they should be able to help him be more disciplined.

### Reciprocating the sacrifice

Reciprocating the sacrifice describes how a family adapts and enacts filial piety. Mrs. P was a 55-year old Korean American who took care of 78-year-old mother-in-law who was diagnosed with dementia. She shared this responsibility with her husband’s two brothers and their wives. During the cold Korean winter, the mother-in-law was sent to California where Mrs. P and her husband resided. Mrs. P explained that her mother-in-law sacrificed a great deal for her three sons and now, the sons were returning the favor by caring for her and by not institutionalizing her. Mrs. P confided that taking care of her mother-in-law was initially “an obligation that she really hated”. She resented the fact the she had to care for an in-law instead of her own parents.

Mrs. P: When I was young, I didn’t like the fact that a woman had to take care of her in-laws, not her own parents. When you had a son, you were kind of insured. And I just did not like it.

As Mrs. P grew older, however, she empathized with her mother-in-law. In their shared history and common life trajectory, Mrs. P saw herself.

Mrs. P: I feel sad by looking at her sometimes. I remember her when she was at my age. She was full of energy and very loving. She still is. It won’t be long before I get her age. When you become my age, then, you will understand. Nobody knows the future. Nobody expected she would end up like that, relying on her sons taking care of her.

For Mrs. P, her dedication had a bittersweet twist when she realized that, cultural changes being what they are, she likely would not receive the same kind of care from her own children.

Mrs. P: I cannot ask my children to take care of me. In my generation, it was how things were. So I didn’t have to think about it. Younger generation now… they are different. I don’t know if my son will take care of me. They say once you have a child, you will understand your parents. Who knows if my son will really understands me. And even though they do, their wives probably will not.

Mrs. P felt that sharing the caregiving responsibility improved her relationship with her extended family members, especially with her sister-in-laws. Because those sister-in-laws lived in Korea and Mrs. P in California, she did not have a close relationship with them. But after the family meeting 4 years ago, the sisters-in-law talked more often and longer, and their emotional bond grew. “We talk out of practical necessity as well as emotional needs”, Mrs. P reported.

## Discussion

This paper described three family caregiving patterns of Korean Americans and the findings of this study warrant further study to examine the influence of Confucianism among Korean American families. This paper highlights the parent–child relationship, which is grounded on Confucian principles of mutual and reciprocal obligations [48]: Parents provide for their children’s care and education; children practice filial piety, the responsibility to respect and care for their parents. Study participants have shown varying ways to organize their life to meet these obligations according to their own concerns and context. Some parents managed their child’s illness by attempting to limit stressors (insulating from the outside world); others emphasized their child’s education so that he or she could be self-reliant (prioritizing education over well-being). These variations may explain the complexities embedded in a culturally dissimilar family with a mentally ill member. To make matters more complex, the family caregiving patterns presented in this paper are not mutually exclusive, although they were presented as distinct categories. For example, although Mrs. K tried to limit the stressors on her daughter, she expressed deep sorrow that her daughter had to drop out of an Ivy League university because of her mental illness. Her daughter’s unrealized potential reinforced Mrs. K’s determination to provide her daughter with a sanctuary, but also increased her concerns about her daughter’s independence if she preceded her in death.

Identifying different patterns of family coping will contribute to mental health nursing practices in several ways: (a) anticipating the needs of Korean Americans with a mental illness and adapting clinical practices for this population, (b) providing a benchmark for family assessment and for hypothesizing about the family dynamics, and (c) developing individualized, culturally appropriate, family interventions in the most effective manner possible. For example, in families reciprocating the sacrifice, assessing how extended family members arrange the caregiving responsibilities is important. Similarly, nurses may have to communicate with multiple family caregivers who reside in different places, including overseas.

Elderly Korean patients who are placed in a long-term care facility may feel ashamed because it implies that they failed to raise a good child who honors filial piety. Likewise, families who place an ill family member in an institution may feel guilty. Thus, nurses who work with this population should be mindful of the emotional distress families and patients may feel and provide them with appropriate emotional support.

Families who try to insulate the family member with mental illness may be reluctant to engage social services for fear of labeling him or her. Thus, nurses must be sensitive to this concern when suggesting social support to these families. To gain family trust and maintain its support, consistent interaction with a small number of health care providers may be an effective strategy. Conversely, families who prioritize education over the well-being may be more receptive to social services and placing a family member in professional care, if such services are presented in the context of education. Because these families may be emotionally overburdened, it becomes important to assess available social support for the family and the caregiver’s ability to adequately manage his or her own stress.

Few studies have investigated family caregiving with Confucianism as the theoretical underpinning. Similar coping styles to those discerned in this study were observed in the literature on chronic illness management [49]. Families coping with chronic illnesses may share common characteristics [50,51], although practical challenges of disease management may vary. Further studies are needed to examine whether the diagnosis of a mentally ill family member affects a family’s management style.

The centrality of church in Korean American communities has been well documented [52]. Our data seems consistent with the literature. In this study, two single mothers received significant emotional and instrumental support from church. This support from church may supplement or compensate for the lack of social support from other sources.

Although findings of this study may apply to several ethnic groups who espouse Confucianism, such as people of Chinese, Japanese, and Vietnamese descent, comparative studies are needed to identify nuanced differences and variations among various subgroups to understand how culture affects health and illness. Furthermore, the patterns of caregiving discerned in our data may be emblematic of general familial relations among Korean Americans.

As mentioned in the Methods section, this study employed a two-stage recruitment strategy. Examining the feasibility of recruitment strategies and analytical framework helped further fine-tune research questions and analytic focus. Focused research questions and an analytic framework reduced the need for recruiting large number of participants.

### Limitation

Although well-justified (see methods section and discussion section), the small number of participants may have limited the variations in caregiving patterns. Also the Korean American family caregiving patterns described here need fuller exploration because of the participants’ homogeneity. All participants were immigrant women. To insure interpretive sufficiency, future research should include diverse family caregivers (e.g., fathers, sons, siblings, and spouses), male caregivers, and family caregivers at different levels of acculturation. More focused analyses are needed to understand how the duration of engaging in caregiving impact on caregiver stresses, their practices, and how caregiving activities are organized. The perspectives from various stakeholders of mental health, such as family care receivers and health care providers, are needed to better understand family caregiving practices, which are complex and multifaceted.

## Conclusion

Confucian-inspired family processes of parental/filial obligation may play an important role in Korean American family life. Nurses must be mindful of these underlying cultural mandates of family caregiving when caring for Korean American patients. Also, because diverse families organize and reshape their world creatively to provide care for a member with mental illness, Nurses must be sensitive, respectful, and flexible in meeting families’ needs.

## Competing interest

The author declares that she has no competing interest.

## Authors’ contributions

MP has carried out designing, collecting, analyzing data, and preparation of the manuscript.

## Pre-publication history

The pre-publication history for this paper can be accessed here:

http://www.biomedcentral.com/1472-6955/11/28/prepub
